# GATA family transcriptional factors: emerging suspects in hematologic disorders

**DOI:** 10.1186/s40164-015-0024-z

**Published:** 2015-10-06

**Authors:** Juehua Gao, Yi-Hua Chen, LoAnn C. Peterson

**Affiliations:** Department of Pathology, Northwestern University Feinberg School of Medicine, 251 E. Huron Street, Chicago, IL 60611 USA

**Keywords:** GATA, Transcription factor, Hematologic disorder

## Abstract

GATA transcription factors are zinc finger DNA binding proteins that regulate transcription during development and cell differentiation. The three important GATA transcription factors GATA1, GATA2 and GATA3 play essential roles in the development and maintenance of hematopoietic systems. GATA1 is required for the erythroid and megakaryocytic commitment during hematopoiesis. GATA2 is crucial for the proliferation and survival of early hematopoietic cells, and is also involved in lineage specific transcriptional regulation as the dynamic partner of GATA1. GATA3 plays an essential role in T lymphoid cell development and immune regulation. As a result, mutations in genes encoding the GATA transcription factors or alteration in the protein expression level or their function have been linked to a variety of human hematologic disorders. In this review, we summarized the current knowledge regarding the disrupted biologic function of GATA in various hematologic disorders.

## Background

Hematopoiesis is a finely modulated process controlled by numerous transcriptional and signaling factors. GATA is one of the transcription factors that play an essential role during hematopoietic development. All members of the GATA family have highly conserved DNA-binding proteins that recognize the motif WGATAR through two zinc fingers [1]. The two zinc fingers bind to separate target sites and each has a distinct function. The C terminal zinc finger binds to the GATA consensus sites, whereas the N terminal zinc finger promotes the interaction between GATA and specific DNA sequences through stabilizing the association with zinc finger protein cofactors [1, 2].

Three members of the GATA family of transcription factors are involved in distinct and overlapping aspects of hematopoiesis, GATA1, GATA2, and GATA3. GATA1 is essential in the development of particular hematopoietic cell lineages. The expression of GATA1 on hematopoietic stem cells, common myeloid or lymphoid precursor induces megakaryocytic and erythroid commitment and simultaneously prevents granulocyte-monocyte and lymphoid development. In addition to erythroid cells and megakaryocytes, high level of GATA1 protein expression is also present on mast cells and eosinophils, suggesting a possible role in the terminal differentiation of these cells [3, 4]. The instructive effect of GATA1 on megakaryocytic and erythroid commitment is through interaction with other transcription factors on target cells [5]. The interaction of GATA1 with N terminal zinc finger cofactors such as FOG-1 (Friend of GATA) is essential for megakaryocyte or erythroid development [6]. Concomitantly, the cofactors essential for granulocyte-monocyte and lymphoid commitment such as PU.1, PAX5 and IL-7 are downregulated [7, 8]. Additionally, GATA1 is directly involved in the survival of the erythroid precursors, though activation of erythropoietin receptor (EPO) signaling [9, 10]. GATA1 activates target genes involved in cell cycle regulation or proliferation and differentiation [11, 12].

GATA2 is highly expressed in hematopoietic stem cells, multipotent hematopoietic progenitors, erythroid precursors, megakaryocytes, eosinophils, and mast cells [13–15]. GATA2 is required for proliferation and survival of early hematopoietic cells and mast cell formation, but dispensable for the erythroid and myeloid terminal differentiation [13]. Interestingly, GATA2 gene is one of the target genes GATA1 regulates. In the absence of GATA1, GATA2 can bind to a region upstream of its own promoter and result in histone acetylation and activation of transcription. Upon induction of GATA1 expression, GATA1 displaces GATA2, a process called GATA switch. The decline of GATA2 and the beginning of GATA1 expression contribute to the erythroid commitment and differentiation [16, 17].

GATA3 is essential for multiorgan development and regulates tissue specific differentiation. GATA3 mutation has been previously reported in a developmental syndrome of hypoparathyroidism, deafness, and renal dysplasia (HDR syndrome) [18]. Interestingly, same mutations that abrogate the DNA-binding ability of GATA3 are also present in a subtype of human breast carcinoma [19, 20]. In hematopoietic cells, GATA3 is expressed mainly in maturing and mature T cells and natural killer cells, and plays an essential role in T lymphoid cell development and immune regulation [21–23]. There is evidence that GATA3 is also expressed in multipotent hematopoietic stem cells (HSCs) and regulates the balance between self-renewal and differentiation in hematopoietic stem cells [24, 25].

The molecular mechanism underlying GATA transcriptional factors has been elucidated from numerous studies from cloning of the GATA factors and functional analysis from knockout embryonic stem cells and mutant mouse strains. Genetic studies in families with hematopoietic disorders, particularly with the most recent advances in large scale genetic analysis, provide a comprehensive approach in characterizing the functional role of GATA transcriptional factors in human disease. In this review, we highlight the recent understanding of GATA transcriptional factors and their roles in the various aspects of hematologic disorders.

## GATA1: from leukemia to anemia

**Fig. 1:**
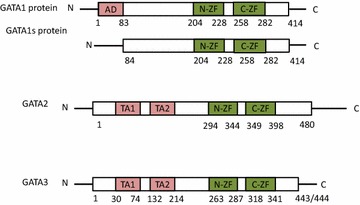
The full length GATA1 protein contains a solitary “N terminal activation domain” (*AD*) and the two Zinc finger domains (*N-ZF*, *C-ZF*). The N terminal zinc finger interacts with cofactor FOG1 and increase the binding affinity to complex DNA motifs. The C terminal zinc finger binds to specific DNA motif “WGATAR”. The short isoform GATA1 protein is the transcriptional product from the shorter splice variant which results in the absence of “N terminal activation domain”. GATA2 and GATA3 encode full length proteins contain two transactivation domains (*TA1* and *TA2*) which contain binding sites for other proteins such as transcription coregulators. The N-terminal Zn finger (N-ZF1) is known to stabilize DNA binding and interact with other zinc finger proteins, whereas the C-terminal Zn finger (*C-ZF*) binds DNA

Mutations in the GATA1 N-terminal activation domain and the N-zinc finger have been linked to human disease  (Fig. 1). Acquired mutations in GATA1 are tightly associated with acute megakaryoblastic leukemia (AMKL) and transient abnormal myelopoiesis (TAM) in children with Down syndrome (DS) [26, 27]. TAM is an abnormal myeloid proliferation that occurs in ~10 % DS newborn. TAM has clinical and morphologic findings indistinguishable from acute myeloid leukemia (AML) but tends to resolve spontaneously without chemotherapy. But about 20–30 % of TAM will develop AML usually AMKL within 3 years. Acquired somatic mutations of GATA1 have been consistently detected in nearly all Down syndrome TAM and AMKL cases [26]. In normal circumstance, both the full length 50KD GATA1 protein product and a 40KD minor isoform are produced. Mutations of GATA1 in TAM and AMKL are clustered in exon 2 and result in a truncated GATA1 protein from a premature stop codon that lacks the N-terminal activation domain. The truncated GATA1 protein interacts with cofactor FOG1 as the full-length GATA1, but with a reduced transactivation potential [26]. The impaired production of full-length GATA1 causes the proliferation seen in TAM and blocks differentiation in AMKL. Screening of the GATA1 mutation fails to detect any mutation from 12 to 25 weeks gestation fetal liver, indicating the GATA1 mutation occurs late in trisomy 21 fetal hematopoiesis [28]. However, GATA1 mutation appears to be the initiating events in the Down syndrome leukemogenesis [29]. Although the risk factors for the progression from TAM to AMKL in DS are unidentified, accumulating evidence suggest the development of AMKL is likely a multistep process; additional genetic events may be required in addition to the GATA1 mutation to develop frank disease [30, 31]. For example, TP53 mutation has been reported present in 2 of 3 patients with DS-AMKL but not in 7 patients with TAM [32]. The type of the mutation and the quantity of the mutant GATA1 protein may also have an effect on the risks of developing acute leukemia, although these observations have not been confirmed in a prospective study with a large series of TAM patients or found to be reliably predicting progression [33, 34].

Mutations in the GATA1 gene have been associated with X-linked familial dyserythropoietic anemia and/or thrombocytopenia. Nichols et al. first described hereditary dyserythropoietic anemia and thrombocytopenia in a pedigree that was consistent with an X-linked disorder. Genetic analysis of GATA1 from available family members revealed a heterozygous G>A mutation in exon 4 which codes for the N-terminal zinc finger domain resulted in a substitution of methionine for valine at amino acid 205 of GATA1 [35]. The V205M mutation impairs the interaction between GATA1 and FOG1, which is essential for both megakaryocyte and erythroid development. This mutation causes skipping of exon 2 and results in loss of long isoform of GATA1 [26]. Several other reports described families with X-linked macrothrombocytopenia, dyserythropoiesis and congenital erythropoietic porphyria harbor mutations in the same zinc finger of GATA1 [36–41] (Table 1). The majority of these mutations involve the N terminal zinc finger domains and cause amino acid changes in the otherwise highly conserved domain. As a result, these mutations adversely affect the binding of FOG 1 to the N zinc finger mutants with a weaker affinity compared to the wild-type GATA1 [36]. The interaction GATA1 and its cofactors are important in megakaryocyte development [42], as the GATA1 recognition site is present in promoter sites for many megakaryocyte-expressed genes [43, 44].

**Table 1:** Reported GATA1 mutations in Diamond–Blackfan anemia, X-linked macrothrombocytopenia and related entities

Authors	Mutations	Impaired function	Clinical features
Sankaran et al. [46]	c.220G>C (p.Val74Leu) exon 2 splice site of the GATA1 gene	Loss of the full-length form GATA1	Diamond–Blackfan anemia
Klar et al. [47]	c.220G>C exon 2 of the GATA1 gene	Loss of the full-length form GATA1	Diamond–Blackfan anemia
Holanda et al. [48]	c.332G>C exon 2 of the GATA1 gene	Synthesis of only the short isoform	Anemia and trilineage dysplasia
Parrella et al. [69]	c.2T > C in the initiation codon	Loss of the full-length GATA-1 isoform	Diamond–Blackfan anemia
Nichols et al. [35]	p.Val205Met exon 4 of N-terminal zinc finger domain	Impairs the interaction between GATA1 and FOG1	Hereditary dyserythropoietic anemia and thrombocytopenia
Freson et al. [36]	c.653A>G (p.Asp218Gly) in N-terminal zinc finger domain	Impairs the interaction between GATA1 and FOG1	Hereditary macrothrombocytopenia and mild dyserythropoiesis
Mehaffey et al. [37]	c.622G>T, 623G>C (p.Gly208Ser) in N-terminal zinc finger domain	Impairs the interaction between GATA1 and FOG1	Macrothrombocytopenia and severe bleeding
Yu et al. [38] and Tubman et al. [41]	p.Arg216Gln in N-terminal zinc finger domain	Affect DNA binding, diminishing the ability of the transcription factor to bind GATA binding sites	X-linked thrombocytopenia, absence or paucity of α-granules, thalassemia
Phillips et al. [40]	p.Arg216Trp in N-terminal zinc finger domain	Alters affinity of GATA1 for either FOG-1, or with GATA recognition sites	Congenital erythropoietic porphyria, thrombocytopenia and thalassemia

Mutations involving exon 2 donor splice site of GATA1 gene have recently been reported in patients with clinical features consistent with the current diagnostic criteria for Diamond Blackfan anemia (DBA) or with DBA like features. DBA is a bone marrow failure syndrome characterized by macrocytic anemia as a result of reduced erythroid precursors in the bone marrow. Although the majority of the cases harbor heterozygous loss of function mutations involving ribosomal protein genes, the molecular pathogenesis remains unclear in a subset of cases [45]. Recently, Sankaran et al. identified the GATA1 mutation involving exon 2 splicing site in 2 siblings with DBA using whole exome sequencing [46]. Subsequently they screened 62 DBA patients with no known mutations of ribosomal proteins and identified one additional patient with the same GATA1 mutation. This mutation is characterized by a deletion of one of 2 adjacent G nucleotides that would impair splicing and frameshift of the full-length GATA1 open reading frame, and as a result, favor the production of the minor isoform of GATA1 protein [46]. Additional GATA1 mutations have been reported in other pedigrees associated with clinical features of DBA (Table 1). All these mutations are predicted to impair the production of the mRNA encoding the full-length form [46–48]. Although it is unclear whether GATA1 mutations define a distinct subset of DBA or it is somehow related to ribosomal dysfunction, a recent study published by Ludwig et al. confirmed the decreased GATA1 mRNA translation in hematopoietic cells from patients with ribosomal haploinsufficiency, suggesting an impairment of selective GATA1 translation initiation from reduction of ribosomal protein as the potential pathogenesis in this subset of DBA [49].

## GATA2: a culprit in disguise

Acquired somatic mutations involving GATA2 are not common in sporadic AML cases. It has been reported in a small subset of AML with CEBPA mutation as acquired secondary genetic events [50, 51]. The GATA2 mutational status does not appear to have any prognostic significance in these patients [51]. More recently, germline GATA2 mutations have been implicated in a group of complex clinical entities with overlapping features including familial myelodysplastic syndrome/acute myeloid leukemia (AML), Emberger syndrome (primary lymphedema with MDS), and MonoMAC syndrome characterized by peripheral monocytopenia, B- and NK-cell lymphocytopenia, increased susceptibility to mycobacterium infections and a predisposition to acute myeloid leukemia and myelodysplastic syndrome. Spinner et al. examined 57 patients from 40 different families with GATA2 mutations, and reported a broad spectrum of manifestations including mononuclear cytopenias, infection, myelodysplasia (MDS), and acute myeloid leukemia, deafness, lymphedema [52]. It is not completely surprising as GATA2 is a versatile transcription factor regulating hematopoiesis, immunity, inflammatory and developmental processes. Recent work established GATA2 as a MDS/AML predisposition gene, in addition to the previously reported RUNX1 and CEBPA. GATA2 associated familial MDS/AML have only been described recently, but studies from a dozen pedigrees indicated clear heterogeneity in the clinical features (Table 2). Patients with GATA2 mutation are younger than controls with sporadic MDS/AML and wild-type GATA2. But the onset age of disease in affected family members are variable. Familial MDS/AML may arise without preceding hematologic abnormalities. Disease progression from MDS to AML in patients with GATA2 deficiency appear to be more rapid compare to wide type MDS cases with comparable IPSS scores [53]. Acquiring secondary genetic abnormalities such as ASXL1 gene mutation are considered as important events during progression [54, 55].

**Table 2:** Reported GATA2 mutations in familial MDS/AML

Authors	Mutations	Locations	Clinical features
Hahn et al. [56]	c.1061C>T (p.Thr354Met), c.1063_1065delACA (p.Thr355del)	C-terminal zinc finger domain	Familial MDS/AML
Bodor et al. [70]	c.1061C>T (p.Thr354Met)	C-terminal zinc finger domain	Familial MDS/AML
Holm et al. [58]	c.313_314insCC (p.Leu105ProfsX15), c.121C>G (p.Pro41Ala), c.1187G>A (p.Arg396Gln), c.1061C>T (p.Thr354Met)	Various regions	Familial MDS/AML, lymphedema, skin cancer
Pasquet et al. [71]	c.1187G>A (p.Arg396Gln), c.610C>T (p.Arg204X), c.670G>T (p.Glu224X), c.988C>T (p.Arg330X), c.1114G>A (p.Ala372Thr), c.1162A>G (p.Met388Val), and a 61 kb deletion of the GATA2 locus	Various regions	Chronic neutropenia and evolution to MDS/AML
Kazenwadel et al. [72]	c.1061C>T (p.Thr354Met), p.Leu332Thrfs*53, deletion encompassing GATA2 gene, p.Met1del290, c.1017 + 2T>G (p.?)	Various regions	Familial MDS, MonoMac
Gao et al. [73]	p.Thr358Asn, p.Leu359Val	C-terminal zinc finger domain	MDS/AML, immunodeficiency
Fujiwara et al. [74]	p. Arg330X	N-terminal zinc finger domain	MDS/AML, immunodeficiency

Cases of AML with GATA2 mutations are reported demonstrating a spectrum with different morphologic subtypes and variable cytogenetic abnormalities, including most frequently monosomy 7, but also trisomy 8, and trisomy 21 [56]. There is a marked genetic heterogeneity ranging from single base substitutions, deletion, and frameshift mutations, present throughout the GATA2 gene. Two types of GATA2 mutations have been described. Mutations occur in C-terminal zinc finger domains interfere the interaction with DNA, other transcription factors and cofactors, and leads to more variable phenotypic consequences. The N-terminal frameshift mutations result in a nonfunctional protein lacking most of the function of the C terminal [57, 58]. Development of secondary mutations, which may occur at different times for affected individuals, may also contribute the heterogeneity in the clinical manifestation. Patients with familial MDS/AML associated with GATA2 mutation have increased risks for severe infections, particular intracellular organisms. AML with GATA2 mutation usually have a poor outcome due to comorbidities such as propensity of infections. Anecdotal cases reported allogeneic hematopoietic stem cell transplant may be beneficial as in addition to eradicating the abnormal myeloid clone, it also offers the benefits to reconstitute the deficient immune cells and correct the propensity for infection. However, the indication or timing of transplant as well as the conditioning regimen and donor source are still being investigated in clinical trials. As there is increasing clinical awareness, and the genetic testing is becoming more available to the clinical laboratories, the incidence of AML with hereditary gene mutations may appear on the rise in the coming years. The unique clinical features may warrant AML with GATA2 mutations, along with other AML with hereditary mutations, to be recognized and treated as distinct entities.

GATA2 mutations have been identified in acute myeloid transformation of chronic myeloid leukemia. Zhang et al. reported a p.Leu359Val in 8 of 85 cases of CML in blast crisis and associated with myelomonoblastic features and a 6 amino acid in-frame deletion spanning the C-terminal border of ZF1 detected in one patient at myeloid crisis with eosinophilia. The p.Leu359Val has a gain of function effect with increased transactivation activity of GATA2 but also enhances its inhibitory effect on the activity of PU.1, a major transcription factor for myeloid cell differentiation [59].

Altered GATA2 protein expression levels by mechanisms other than GATA2 mutations may also be a significant event in leukemogenesis. A recent study by Celton et al. using RNA sequencing reported a reduction in GATA2 protein expression in normal karyotype AML due to aberrant DNA methylation [60]. Along with previous observation GATA2 being one of the most differentially hypomethylated locus in DNMT3a knockout mice [61], these findings implicated the epigenetic regulation of GATA2 is likely, though not sufficient by itself, included in the epigenetic modulation during leukemogenesis [62].

## GATA3: beyond the T cells

GATA3 expression, as an important downstream event of Notching signaling, is required for producing early T-lineage progenitor cells [22, 63]. Sequencing data identified GATA3 mutation as one of the recurring somatic genetic abnormalities in early T cell precursor acute lymphoblastic leukemias (ETP-ALL) with a frequency of approximately 10 % (6 of 64 cases) in a large series published by Zhang et al. GATA3 mutation was not present in any of the 42 non-ETP acute lymphoblastic leukemia [64]. Four of the six cases reported were at R276 residue, which was also mutated in HDR [65]. Most of the mutations were biallelic due to either mutations involving both alleles or concomitant deletion of the second allele, and impair the DNA-binding affinity of GATA3 for its DNA targets and result in loss of GATA3 function [64].

Beyond the commitment to early T cell lineage, GATA3 promotes the development of CD4 + Th2 cells. High expression of GATA3 identifies a biologically distinct subgroup in peripheral T cell lymphoma associated with overall poor prognosis [66, 67]. The gene expression profile of the GATA3 subset of peripheral T cell lymphoma also identifies high expression of Th2 associated transcripts. This observation provides insight in understanding the pathogenesis and potential oncogenic pathways for the peripheral T cell lymphoma. Surprisingly, aberrant expression of the T-cell transcription factor GATA3 is observed in B cell-derived Hodgkin Reed-Sternberg (HRS) tumor cells. The dysregulated GATA3 expression is likely due to constitutive binding of NFkB and Notch-1 pathways to GATA3 promoter elements [68]. The dysregulated GATA3 expression correlates with regulation of IL-5, IL-13, STAT4, and contributes to the complex cytokine and signaling network involving HRS. The role for GATA3 beyond T cell development still needs to be elucidated.

## Conclusion

GATA family transcription factors play essential roles during normal hematopoiesis. Mutations in genes encoding the GATA transcription factors have been linked to a variety of human hematologic disorders. In this review, we summarized recent understanding of how the disrupted biologic function of GATA may contribute to the hematologic diseases. Much of the knowledge regarding the role of GATA transcriptional factors in human hematologic disorders has just started to emerge, but accumulating data indicate their versatile and essential functions in many aspects of hematopoietic system. Some of these findings are rapidly transforming our current view of several hematologic entities.
